# Spatial modelling of healthcare utilisation for treatment of fever in Namibia

**DOI:** 10.1186/1476-072X-11-6

**Published:** 2012-02-15

**Authors:** Victor A Alegana, Jim A Wright, Uusiku Pentrina, Abdisalan M Noor, Robert W Snow, Peter M Atkinson

**Affiliations:** 1 Malaria Public Health & Epidemiology Group, Centre for Geographic Medicine Research - Coast, Kenya Medical Research Institute/Wellcome Trust Research Programme, P.O. Box 43640, 00100 GPO Nairobi, Kenya; 2 Centre for Geographical Health Research, Geography and Environment, University of Southampton, Highfield Southampton SO17 1BJ, UK; 3 National Vector-Borne Disease Control Programme, Ministry of Health and Social Services, Private Bag 13198, Windhoek, Namibia; 4 Centre for Tropical Medicine, Nuffield Department of Clinical Medicine, University of Oxford, CCVTM, Oxford OX3 7LJ, UK

**Keywords:** Namibia, Fevers, Treatment, Spatial, Utilisation, Malaria

## Abstract

**Background:**

Health care utilization is affected by several factors including geographic accessibility. Empirical data on utilization of health facilities is important to understanding geographic accessibility and defining health facility catchments at a national level. Accurately defining catchment population improves the analysis of gaps in access, commodity needs and interpretation of disease incidence. Here, empirical household survey data on treatment seeking for fever were used to model the utilisation of public health facilities and define their catchment areas and populations in northern Namibia.

**Method:**

This study uses data from the Malaria Indicator Survey (MIS) of 2009 on treatment seeking for fever among children under the age of five years to characterize facility utilisation. Probability of attendance of public health facilities for fever treatment was modelled against a theoretical surface of travel times using a three parameter logistic model. The fitted model was then applied to a population surface to predict the number of children likely to use a public health facility during an episode of fever in northern Namibia.

**Results:**

Overall, from the MIS survey, the prevalence of fever among children was 17.6% CI [16.0-19.1] (401 of 2,283 children) while public health facility attendance for fever was 51.1%, [95%CI: 46.2-56.0]. The coefficients of the logistic model of travel time against fever treatment at public health facilities were all significant (p < 0.001). From this model, probability of facility attendance remained relatively high up to 180 minutes (3 hours) and thereafter decreased steadily. Total public health facility catchment population of children under the age five was estimated to be 162,286 in northern Namibia with an estimated fever burden of 24,830 children. Of the estimated fevers, 8,021 (32.3%) were within 30 minutes of travel time to the nearest health facility while 14,902 (60.0%) were within 1 hour.

**Conclusion:**

This study demonstrates the potential of routine household surveys to empirically model health care utilisation for the treatment of childhood fever and define catchment populations enhancing the possibilities of accurate commodity needs assessment and calculation of disease incidence. These methods could be extended to other African countries where detailed mapping of health facilities exists.

## Background

Understanding population health care utilisation and defining the catchment sizes of health providers are important for efficient planning and resource allocation [1,2]. Utilisation is a function of access to health services and is affected by geographical accessibility, alongside many other factors [3-9]. In low income countries, such as those of the African continent where the burden of ill health is greatest [10-14], adequate information on the location of populations, health services, facility workload, patient addresses and socio-demographic characteristics are rarely available to develop high resolution utilisation models nationally [15,16]. Available data on health care utilisation are mainly from routine national household surveys undertaken every 3 to 5 years [17], while few countries have a spatial database of health service providers [18,19]. Recent developments in high spatial resolution population mapping in Africa [20] provide opportunities for understanding the location of services in relation to populations. There are no previous attempts, however, that combine routine household survey data on treatment seeking, such as the MIS, with health facility and population maps to develop spatial utilisation models at the national level in Africa.

Previous approaches to quantifying geographical accessibility to healthcare have included straight-line (Euclidean) distances [19,21-26], drive times [8,27,28] and network analyses [26,29]. Euclidean distances fail to account for different patterns of service use and topography and assume utilisation rates are uniform within facility catchments and that patients always use the nearest facility [6,25,26]. Drive times have been shown to be a preferable measure in developed countries [30] where vehicular transport is widespread, but are unlikely to be useful in a developing country context where a large proportion of the population walk to the nearest facility [26]. An alternative, the cost surface based on travel times, showed closer agreement with the pattern of use in rural South Africa when modelled as a logistic function [31]. These forms of distance measurement have been used to analyse utilisation by using metrics such as number of health facilities within a certain predefined distance of the facility, the average distance to *n *number of health facilities and the gravity model [6,32]. The gravity model is a spatial interaction model analogous to Newton's law of gravity where the force of attraction between two bodies varies proportionally to the product of their masses and inversely to distance between them [33,34]. In this form, patient interaction with healthcare is denoted by flow from patient origin to the health service while the masses are represented by various utilization effects such as cost, size of health facility or propensity of patient groups to use healthcare [15]. The other distance metrics either ignore the interaction with other possible providers within the considered region or may assume that patients always use the nearest facility [35].

In this study, utilisation of the public health sector in Namibia was modelled using information on source of treatment of fevers from a routine national household survey. First, theoretical surface of travel times to nearest health facility were developed and combined with reported treatment seeking patterns to predict probability of using a facility on a 1 km by 1 km grid. The theoretical surface of travel times was used to define spatial catchments around public health facilities while the predicted probability of use was used in combination with population distribution and fever burden to define cases unlikely to seek care from the public health sector.

## Methods

### Health system in Namibia

Namibia is divided into 13 regions (administrative level 1 boundaries) and 34 health districts. Health care services are provided by the government (more than 70%), mission or faith-based facilities, and the private sector [36,37]. The mission facilities are predominantly located in rural areas. The health system is decentralised with regional-based directorates overseeing management at district levels [36,38,39]. The country is sparsely populated with some regions having very low population density [38]. The government of Namibia allocates about 12% of the national budget to health care [40]. Of the total expenditure on health, over 60% is aimed at inpatient and outpatient care.

### Datasets

#### Healthcare facilities

In an effort to understand service provision and improve resource allocation, the Namibia government, with support from multilateral agencies, conducted a facility census in 2009 [37]. During the census, facilities were classified by level of service provision and provider with hospitals ranked as the highest level followed by health centres, clinics and sick-bays. The majority of these facilities were mapped using global positioning system (GPS) receivers. For those that had not been positioned using GPS, coordinates were derived through geocoding of place or village names, using reference settlement datasets and online sources such as Google Earth [41] and Geo-names [42].

#### Population distribution

The Namibia population surface from Afripop [20] was used for this analysis. It was developed from a combination of census and land cover data using dasymetric techniques [43]. Dasymetric methods involve the decomposition of census data to improve their spatial resolution [44]. A detailed description of this population map layer is provided in Linard et al. [41] and is also available at http://www.afripop.org/. In brief, land cover map from MEdium Resolution Imaging Spectrometer (MERIS) (GlobCover) was combined with fine spatial resolution data on settlements to produce a population layer in raster format [45]. Settlements data for Caprivi, northern-central and Kavango regions were obtained from the environmental atlas project [46-48] while an estimation of urban population in Windhoek was based on water demand report from water resources management review and included information from the census of 1991 [49]. GlobCover was originally provided at a spatial resolution of 300 m and its land cover classification is compatible with the UN land cover classification system (LCCS) [50]. During the production of the population layer, a finer land cover or land use layer was created to include detailed information on roads, rivers and settlements extents. The resulting classes were then assigned a weight value calculated based on density. The value was then used to re-distribute population polygon data in 1 km by 1 km spatial pixels [45]. The resulting national population map was then projected forward to 2010 using the United Nations' (UN) urban and rural inter-censual growth rates http://esa.un.org/unup/. The number of children under the age of five years for each region was estimated by multiplying the proportion in this cohort at regional level, from UN national census estimates [51] with the national population map.

#### Healthcare utilisation

Data on the prevalence and attendance at a health facility for treatment of fever at household level among children under the age of five years were obtained from the Namibia Malaria Indicator Survey (MIS). This survey was carried out in the endemic malarial areas in northern Namibia, namely the Caprivi, Kavango, Kunene, Ohangwena, Omaheke, Omusati, Oshana, Oshikoto and Otjozondjupa regions from April to June, 2009. It was based on a random two-stage cluster sampling design in which clusters were first sampled within each region and, within each cluster, households were sampled randomly [52]. A cluster in the MIS consisted of approximately 25 households that were mapped using GPS. The tools used to carry out the MIS were developed by the Monitoring and Evaluation Working group (MERG) of the Roll Back Malaria (RBM) programme and include questionnaires, manuals and guidelines based on the Demographic Health Surveys (DHS) [52,53].

#### Ancillary data

A roads layer was downloaded from a freely available online archive [54] while hydrological data were obtained from the digital atlas of Namibia [55]. Elevation data set, the Advanced Spaceborne Thermal Emission and Reflection Radiometer-Global Digital Elevation Model (ASTER-GDEM) was downloaded from online resources available at http://asterweb.jpl.nasa.gov/gdem-wist.asp. ASTER-GDEM was released in 2009 jointly by NASA and Japan's Ministry of Economy, Trade and Industry (METI). The elevation data have a spatial resolution of 30 m (0.000277° by 0.000277°) and are archived using 1° by 1° tiles in GeoTIFF format. Downloaded tiles were mosaiced in ArcGIS (ESRI, Redlands, CA, version 10). A land cover surface for 2009 was obtained from the MERIS GlobCover product http://ionia1.esrin.esa.int/[56] at 300 m spatial resolution.

### Analysis

#### Developing a surface of travel time to public health facilities

A raster surface of travel times between facilities and population grid squares was generated using a combination of land cover (GlobCover), elevation, road and river layers in AccessMod (version 3.0) [57]. In deriving this surface, each GIS layer was converted into a raster surface at 1 km by 1 km and each pixel was assigned an impedance value. The resulting raster layers were then combined into a single *'cost' *grid based on cumulative travel speeds. The modeled travel times were chosen as the best representation of access because (1) people readily relate to travel time to health facility compared to physical distance and a question on travel time to facility is included in MIS surveys and (2) travel times are more comparable across different countries. Travel speeds were assigned to different land cover types, roads and slope by assuming multiple modes of transport within a single journey to a health facility. For the primary and secondary roads, motorized transport was assumed because a simulation of both motor transport and walking on the same route requires a more complex algorithm. Speed on these roads was assigned based on the Namibia national guidelines for roads [58]. For tertiary roads, a correction for non-motorized transport (cycling) at 10 km hr^-1 ^was applied [57]. It is possible that a walking correction can also be applied to the tertiary roads, but this would also require a complex representation to simultaneously correct for both modes. Ray and Ebener [57] recommended the use of different travel speeds for different land cover or land use classes. Land cover classes were assigned travel speeds (Table 1) based on these recommendations and from previous studies [26,31]. The maximum walking speed assigned to tree cover class, sparse vegetation and shrubs was 5 km hr^-1^, while for desert landscapes a 2 km hr^-1 ^speed was used. Slope was derived from elevation data using the ArcGIS (ESRI, Redlands, CA, version 10) raster analysis tools and different speeds calculated for each degree rise based on Tobler's equation (*V *= 6*exp(-3.5 abs[Tan(slope in degrees/57.296) + 0.05]) [59] where *V *is the calculated speed. Hence, on flat terrain, the walking speed is about 5.0 km hr^-1 ^while for a 20° rise in slope, the speed is lower (1.4 km hr^-1^).

**Table 1:** Description of various data and their sources used as input into the development travel time to the public health facilities in northern Namibia.

Map Layer	Description	Classification	Speed (km/h)	Model
Land use/land cover	Spatial representation of all different land use and land cover types. Two land cover grids were processed (1) basic land cover grid (2) combined grid that incorporates roads and rivers with same resolution as the DEM	Tree cover, broad leaved deciduous or evergreen	5	WALKING
		Tree cover, needle leaved, deciduous or evergreen	5	WALKING
		Tree cover, other	2	WALKING
		Shrub cover	5	WALKING
		Herbaceous cover	3	WALKING
		Sparse herbaceous	4	WALKING
		Cultivated and managed areas	5	WALKING
		Bare areas/desert	2	WALKING
		Water Bodies	0	NONE

Roads	Classified into three broad categories; Primary roads (class A), Secondary roads (class B); Tertiary roads (class C). Each road class was assigned a slightly different speed.	Primary roads	80	NONE
		Secondary roads	60	NONE
		Tertiary roads	10	CYCLING

Rivers	GIS layer representing barrier to movement. Only major rivers were used to reduce the complexity of running the algorithms	NA^1^	0	NA^1^

Digital elevation model	Altitude values that are used in anisotropic calculation; Original DEM 30 m ASTER grid; resampled to 100 m pixel size	Degree of Slope (< 0.5°)	4.88	WALKING
		Degree of Slope (5.0°)	3.71	WALKING
		Degree of Slope (10.0°)	2.71	WALKING
		Degree of Slope (20.0°)	1.41	WALKING
		Degree of Slope (30.0°)	0.66	WALKING

The assumed travel speeds for each input feature are also shown

1. NA is an abbreviation for ' Not Applicable'

#### Fitting distance decay models

Travel times to the nearest facility were calculated for each household in the MIS survey (*n *= 2,823). First, to evaluate the potential impact of using travel times rather than a simpler distance measure, travel times were compared to straight line distance, derived in ArcGIS (ESRI, Redlands, CA, version 10), between household and the health facility. Pearson's correlation coefficient was estimated to assess the association between computed times and straight line distance. Second, a logistic model of the form *Y = C/(1 + e^(A-x)/B^) *[60] was fitted to measure the effect of theoretically derived travel times on attendance at a facility, with attendance versus non-attendance at a facility as the dependent variable and travel time as the explanatory variable. The model has three coefficients: *C*, a limiting function on the y-axis that measures probability of attendance when distance is zero; *B*, a distance decay parameter; and *A *an asymptote factor at an inflection point of the model. A goodness-of-fit statistic, *t*, was examined for each estimated coefficient along with the *p*-values. A model representing the decay in probability of attendance with travel time was fitted to the survey data.

#### Defining health facility catchments and catchment population for children (0-4 years)

Catchment areas were calculated for each facility using '*cost' *allocation *Spatial Analysis *tools (ArcGIS 10; ESRI Inc.). Using the travel time surface and limiting the maximum travel time of any facility catchment to 3 hours based on the travel time decay model, catchment areas were modelled. Unlike previous approaches where all individuals were considered to be within the catchment area of a facility [1,25,26,31], this travel time threshold approach produces more realistic catchments areas in which certain populations are outside the reach of a health facility. Population counts for children under the age of five years in each catchment boundary were extracted and multiplied by the probability of attendance for fever treatment to derive the proportion of children likely to attend a public health facility. Population outside any health facility catchment were similarly estimated. Derived population counts were then aggregated by region for comparison.

#### Estimating the burden of fever in the public health sector in northern Namibia

Prevalence of fever among children under the age of five years in the northern Namibia was derived at a sub-national level (administrative 1 boundary) from the MIS and applied uniformly to each health facility's catchment population. The result was an estimated number of febrile under-five children within each facility's catchment. Within each catchment the number of fever cases that will attend the health facility for the treatment of fever was further calculated by multiplying the probability values at each pixel by the number of fever cases at the same pixel. The number of fever cases unlikely to attend the public health sector was finally calculated by subtracting those likely to seek treatment from the total fever burden. It is noted that the proportion of fever cases that seek treatment at a public health facility is likely to be different from overall fever burden [61]. Calculated fever counts by treatment seeking were aggregated and compared at sub-national level.

## Results

### Fever and facility attendance

**Figure 1:**
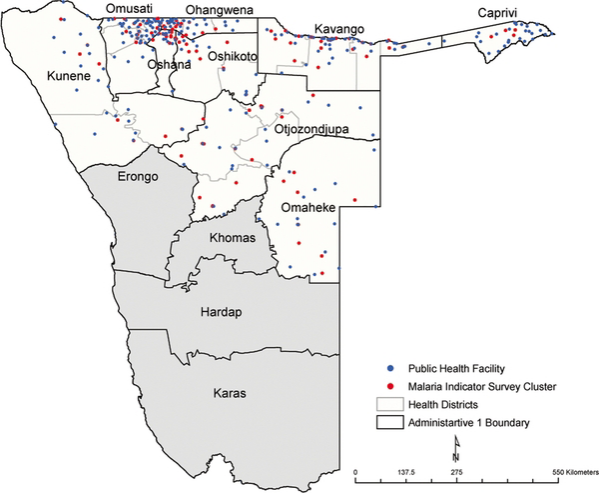
**Location of the 2009 MIS clusters shown as red dots (N = 120) in relation to public health facilities shown as blue dots (N = 245) in the nine northern provinces of Namibia where the 2009 MIS was undertaken (Kunene, Omusati, Oshana, Ohangwena, Otjozondjupa, Omaheke, Kavango and Caprivi)**. Subsequent analysis was restricted to only these nine regions.

In the MIS survey, 13,569 individuals in 120 clusters (Figure 1) were interviewed of which 2,283 (16.8%) were children under the age of five years. Overall, 401 or 17.6% [95%CI: 16.0-19.1] of these children reported to have had at least one episode of fever two weeks prior to the survey (Table 2). Of those who reported to have had fever, attendance at a public health facility was 51.1% [95%CI: 46.2-56.0]. Overall, the proportion of children with fever in the MIS was fairly homogeneous across all the regions surveyed.

**Table 2:** A summary of treatment seeking behaviour for fever among children under the age of five as reported during MIS 2009 undertaken in the northern provinces of Namibia

MIS, 2009					
**Regions**	**Number of Clusters**	**Number of children under five years of age**	**Percent (95% CI) children under five years of age with fever 2 weeks prior to survey**	**Percent (95% CI) of children under five years of who sought treatment**	**Percent (95% CI) children under five years of age with fever in last 2 weeks who sought treatment in public health sector**

Caprivi	6	105	27.6(19.0-36.2)	55.2(36.7-73.6)	48.3(29.7-66.8)
Kavango	28	670	23.9(20.6-27.1)	66.9(59.5-74.2)	57.5(49.8-65.2)
Kunene	9	178	17.4(11.8-23.0)	45.2(27.3-63.0)	41.9(24.2-59.6)
Ohangwena	14	304	11.8(8.2-15.5)	58.3(42.0-74.7)	58.3(42.0-74.7)
Omaheke	9	175	17.1(11.5-22.7)	56.7(38.6-74.8)	53.3(35.1-71.5)
Omusati	14	229	12.7(8.3-17.0)	58.6(40.3-76.9)	51.7(33.2-70.3)
Oshana	12	176	14.8(9.5-20.0)	61.5(42.4-80.7)	53.8(34.2-73.4)
Oshikoto	10	171	7.0(3.2-10.9)	58.3(29.1-87.6)	58.3(29.1-87.6)
Otjozondjupa	18	275	17.5(13.0-22.0)	29.2(16.1-42.2)	27.1(14.3-39.8)

Total	120	2,283	17.6(16.0-19.1)	57.1(52.2-62.0)	51.1(46.2-56.0)

### Model fitting results

**Figure 2:**
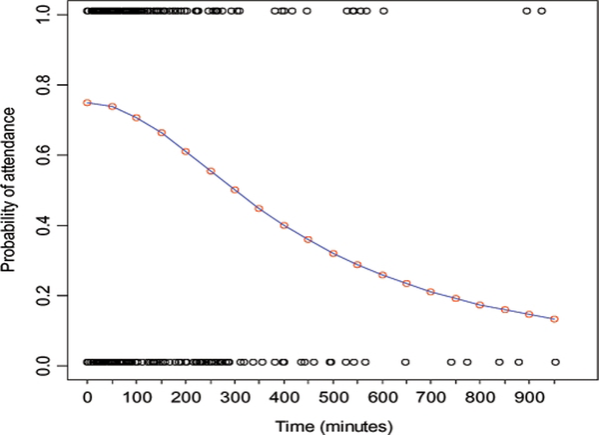
**Probability decay function for the MIS survey showing probability of attendance (*y*-axis) for treatment seeking group against increasing travel times (*x*-axis); *Y = C/(1 + e^(A-x)/B^) *where *C *(0.766) is the limiting function on the *y*-axis; *A *(3.736) is the asymptote factor at the inflection point of the model; *B *(-0.609) is the decay parameter. The model was run using log transformed travel time (*x*-axis) and later back transformed for presentation purposes. The attendance pattern (1 = attendance and 0 = non-attendance) is also superimposed on the decay curve**. The coefficient of all parameters were significant at p < 0.001.

Figure 2 shows the modeled decay curve in utilization with increasing travel time on the *x*-axis for northern Namibia. The curve shows that treatment seeking decays more rapidly after travel times of approximately 180 minutes. The coefficients of the logistic model, fitted for the modelled travel times against attendance of the public health facilities for treatment of fever by children under the age of five years reported during the MIS, were all significant with P < 0.001 (Figure 2). In the model, the residual standard error was 0.020 with the sum of squared residuals equal to 0.005 indicating a good model fit to observed fever treatment patterns

### Probability of attendance at public health facilities

**Figure 3:**
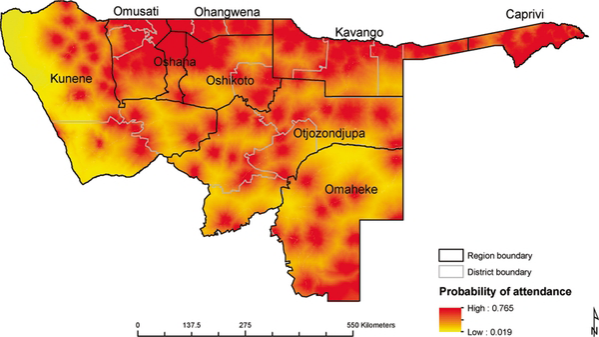
**Map of probability of attendance for treatment fever by children under the age of five years at the nearest health facility based on the MIS 2009**. The map shows the 9 regions in north Namibia where MIS was carried out namely; Kunene, Omusati, Oshana, Ohangwena, Otjozondjupa, Omaheke, Kavango and Caprivi. The lowest probability was 0.02 and the highest probability was 0.76.

**Figure 4:**
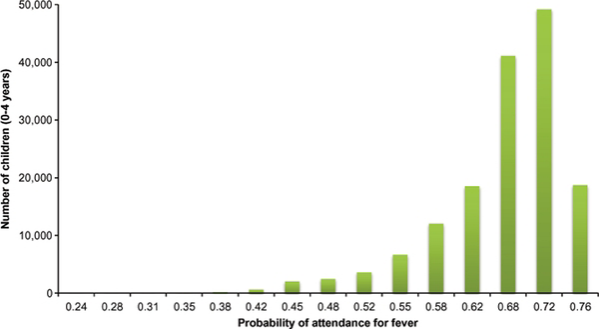
**Number of children under the age of five years (y-axis) against an increasing probability of attendance for fever (x-axis) at the nearest public health facility**. Majority of children were at a probability greater than 0.5 with maximum probability of attendance of 0.76.

**Figure 5:**
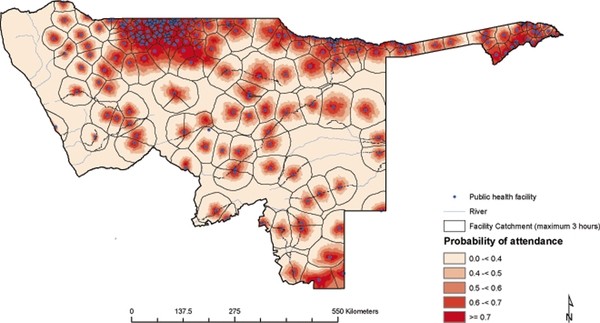
**Map of northern Namibia showing health facility catchment areas developed using the modelled travel time to the nearest public health facility overlaid with the probability of attendance of a public health facility by children less than five years of when sick with fever**. The health facilities are shown as blue dots. Darker shades of red represent increasing probability.

Probability of attendance was estimated and mapped for the northern parts of Namibia based on the utilisation model and the map of health facilities (Figures 3 and 4). The proportion of children likely to seek treatment for - fever in public health facilities was estimated based on probability of attendance at a given travel time. Out of the estimated total of 162,286 children under the age of five years living in northern Namibia, 160,294 (98.8%) children were estimated to live within public health facility catchments (Table 3). Figure 4 shows the distribution of children under the age of five years by probability of attending a public health facility when sick with fever. Although the majority of children (82%) were within distances where the probability of attending a public health facility was ≥ 60% (Figure 4), a minority (4.6%) had much lower probabilities of attendance (less than 0.5) including the few who lived outside of the catchment of any public health facility. The estimated overall fever burden in the north of Namibia in 2009 was 24,830 cases assuming a single episode of fever per child per year (Table 3 and Figure 5). Of the estimated burden, 8,021(32.3%) were within 30 minutes of travel to nearest public health facility and after adjusting for the probability of attendance 8,616 (34.7%) of these cases were unlikely to have been treated in the public sector.

**Table 3:** Estimated number of children under the age of five by province and their modelled treatment seeking for fever at the nearest public health facility

	Estimated number of children under five years of age in 2009	Estimated number of children under five years of age within a PHF^1 ^catchment	Estimated number of fever cases among children under five years of age based on MIS prevalence	Number(Percentage) of children under five years of age with fever likely to attend a PHF^1^	Number(Percentage) of children under five years of age with fever not likely to attend a PHF^1^
**Region**					
Caprivi	8,881	8,741	2,433	1,637(67.3)	796(32.7)
Kavango	20,244	20,374	4,825	3,264(67.6)	1,561(32.4)
Kunene	8,192	7,363	1,425	588(41.3)	837(58.7)
Ohangwena	32,167	30,863	3,793	2,695(71)	1,098(29.0)
Omaheke	11,550	11,051	1,974	1,060(53.7)	914(46.3)
Omusati	27,386	26,993	3,478	2,522(72.5)	956(27.5)
Oshana	14,973	13,088	2,186	1,648(75.4)	538(24.6)
Oshikoto	19,918	23,661^2^	1,395	932(66.8)	463(33.2)
Otjozondjupa	18,977	18,160	3,321	1,868(56.3)	1,453(43.7)
**Travel time**					
< 30 minutes	51,791	51,791	8,021	6,056(75.5)	1,965(24.5)
> 30 minutes - < 1 hour	98,620	98,620	14,902	11,218(75.3)	3,684(24.7)
> 1 - < 2 hours	138,219	138,219	19,035	14,136(74.3)	4,898(25.7)
> 2 - < 3 hours	160,294	160,294	20,799	15,279(73.5)	5,520(26.5)
> 3 hours	1,992	-	4,031	934(23.2)	3,096(76.8)
**Probability of attendance**					
< 0.50	13,698	11,808	2,277	19(0.8)	2,258(99.2)
> 0.50 - < 0.60	7,820	7,908	1,356	717(52.9)	639(47.1)
> 0.60- < 0.70	18,944	18,963	2,928	1,925(65.7)	1,003(34.3)
> 0.70- < 0.75	120,862	121,615	18,269	13,553(74.2)	4,716(25.8)

**Total**	**162,286**	**160,294^3^**	**24,830**	**16,214 (65.3)**	**8,616 (34.7)**

1. PHF is an abbreviation for 'Public Health Facility', which in this case does not include private facilities or privates for profit

2. For Oshikoto region, the estimated number of children (0-4 years) slightly exceeds the overall population estimate for the region. This is because the catchment boundaries in some cases overlap the regional boundaries

3. The total number of children 0-4 years old in catchment boundaries was lower than the total estimated under fives population because of (a) not all children within the catchment were assumed to use the public health facility (b) the catchment boundaries did not covering 100% of the entire population by limiting maximum travel time to 3 hours from the decay model

## Discussion

Data from MIS showed a moderate self-reported use of public health sector (51.1%, 95%CI: 46.2-56.0) for treatment of fever among children under the age of five years northern Namibia. Using this data, utilisation of the public health sector in northern Namibia for the treatment of fevers among children under the age of five years was modelled as a function of travel time. This function was used to model the probability of attendance at a health facility and the number of children likely to attend the nearest facility. Not surprisingly, the probability of attending the nearest health facility for the treatment of fevers was greatest in areas where there was a high concentration of public health facilities and population. Of the estimated 162,286 children under the age of five years in the northern provinces, 160,294 (98.8%) were within a public health facility catchment with overall fever burden of 24,830 cases. The proportion of fevers within the catchment of the public health sector in northern Namibia was 90.8% (22,553) and 16,195 (65.3%) were likely to use the public health sector. This implied that overall, 8,616 (34.7%) febrile children were unlikely to use at a public health facility in northern Namibia including 4,030 (47%) fever cases that lived outside of a health facility catchment (Table 3, Figure 5).

This analysis builds on previous work to define facility catchments with information on utilisation patterns. Approaches similar to the one used in this study were presented in Tanser et al. [31] but using a two parameter logistic model and in Noor et al. [26] using a transect algorithm to characterize facility choice. These studies used local rather than national data sets with Tanser et al. [31] considering all forms of healthcare utilisation, not specifically fever. Whereas our fever-related attendance data suggest not all households used facilities at a travel time of zero, the Tanser et al. [31] study assumed that all households attended the healthcare facilities when the travel time was zero and the distance decay was more rapid than that observed here. This assumption is unlikely because regardless of proximity of households to a given health facility, treatment seeking behavior varies and are driven by other individual, household and community level choices [62]. The model presented here also differs from the two parameter logistic model in that a third parameter was included to represent the phenomenon discussed by Pinheiro and Bates [60] which can be thought of as a generalization of the two parameter model where facility attendance is less than 100% at a travel time of zero. Previously, such models have been common in resource growth applications [63], but not in public health applications such as modeling facility utilisation. The third parameter *C *in the model significantly modifies the behavior of the logistic function. It represents a horizontal asymptote at a limiting value *C *which can be less than 1 (whereas the two parameter model eventually levels off at an asymptote of 1 as it approaches -∞) [64]. This value does not relate directly to the proportion that sought treatment, but represents the maximum probability. Therefore, the logistic function never exceeds this value. The rate of decay of the logistic function is determined by the exponential function, *e^(A-x)/B^*, in the denominator. An additional difference from the approach used here and those of previous studies is the application of a travel time threshold (3 hours) based on the distance decay curve, outside of which individuals were regarded to fall outside the catchment of any public health facility. Finally, to compute the number of fevers that will use a public health facility the continuous probability surface was multiplied with the fever case burden maps with assumption that, at every distance, the probability of using the public health sector will be < 1, thus, some will not seek treatment at a public health facility regardless of proximity or will seek treatment at facilities that is not the nearest. This approach, we believe, models a scenario closer to real-life utilization of health services in many African countries.

The methods used here have some limitations. First, in modeling travel times it was assumed that patients travel to their nearest facility by either walking, using a bicycle or any form of public transport. In contrast, people might not use the closest clinic due to various factors such as choice of better quality provider or income, socio-economic status, individual and community preferences and severity of illness [4,22,26,35,65,66]. However, since the MIS does not identify the specific healthcare facility used by an individual, it is not possible to model such effects from the available data. The exclusion of these determinants into the model are likely to change the probability of attendance computed for each travel time band and may be a reason for higher utilization than that reported in the MIS (14.2% difference). In addition, for various land cover classes an assumption was made that a patient would either (a) walk to their nearest facility with a correction for particular land use or land cover class and slope, (b) use a car as a form of transport to the facility or would walk to the nearest road then use public means of transport for rest of the journey or (c) cycle to the health facility. In reality, the distribution and use of public transport may be more sporadic and less predictable at the national level. Such discrete assumptions, however, are a tradeoff between model complexity and precision and empirical data to test and improve these assumptions are rarely available in sub-Saharan Africa settings. In estimating the fever burden for each facility's catchment, aggregate fever prevalence rates within each region were used. In reality fever prevalence is likely to vary by facility catchment and rates at this level may be considerably different to the regional mean prevalence which masks fine scale heterogeneity. Finally, the analysis focuses only on the northern regions of Namibia where household treatment seeking data are available and where the concentration of health services and population are highest. It is likely that the geographically larger but very sparsely populated southern regions will have different service utilisation patterns. Additional data is needed to model a nationwide estimate of service utilisation, catchment areas and catchment populations.

The relationship between travel time, service utilisation and disease outcomes has been demonstrated in several studies. Moïsi et al. [67] showed increasing travel time (greater than 1 hour) from a hospital was associated with disease severity among children under the age of five years with pneumonia and suspected meningitis. Another hospital case-control study carried out in Yemen illustrated similar outcomes, with children (6 to 10 years) likely to develop severe malaria with increasing distance (greater than 2 km) from the hospital [68] while O'Meara et al. [69] showed that incidences of malaria hospitalisation doubled after 2 hours of travel time from health facility. In these studies, children living at a further distance were likely to wait longer before using a health facility, leading to severity of illness that required in-patient care. Recent findings from a study in Ethiopia [70] suggest a five-fold risk of death (all case mortality) amongst children in rural population at a greater travel time (2 hours) from a nearest health facility. While the effect of travel time or distance has been associated with poor health outcomes, few studies demonstrate treatment seeking patterns within a health facility catchment which in turn are potentially useful in forecasting commodity needs for case management [71], optimization of facility-based interventions [66,67,70,71] and estimating underlying populations to define disease incidence [72]. Okiro et al. [72] defined facility catchments at several hospital sites in Kenya using enumeration areas where over 90% of malaria cases resided, while Gething et al. [66,73] assumed that catchment populations could be linearly related to total case loads observed for a long period of time (over five-years) at a facility and used this to standardize out-patient clinically diagnosed malaria data.

## Conclusion

National representative health sample surveys such as the MIS, DHS, Multiple Indicator Cluster Surveys (MICS) and the AIDS Indicator Survey (AIS), usually constitute a representative sample of the national population and are being used to study health outcomes and impact of interventions in Africa [74-76]. Although the Namibia MIS of 2009 was only undertaken in the northern regions, this study nonetheless demonstrates the potential of routine household survey data on treatment seeking for fevers to model public health care utilisation, catchment areas and populations in Africa. This provides the opportunity to model facility catchment population, compute disease incidence and determine risk and resource needs. This approach could be extended to the large accumulation of DHS, MIS and the MICS surveys conducted by the United Nations Children's Fund in several countries Africa. This possibility is further improved by the availability of high spatial resolution population maps of Africa under the Afripop project [20] and the increasing spatial resolution of ancillary spatial data such as roads, elevation, drainage, land cover and land used data. The input data, however, require improvements in two important ways. First, countries must have maps of all their health facilities or at least of those that are in the public sector. Secondly, even though utilisation patterns appear stable regardless of time of survey, treatment seeking data are available only for children under the age of five for almost all the national household surveys. In a recent MIS undertaken in the 15 northern states of the Sudan, a country with similar ecology and utilisation patterns as Namibia, fever treatment seeking among all ages was recorded showing minimal change in treatment seeking with age [77]. While it is not clear if this holds true for all other African countries, focusing on a narrow age group in areas where fever prevalence is low may result in unstable sample sizes and low model precision. Future surveys should, therefore, explore the possibility of capturing information on a wider age range, preferably for all ages.

## Abbreviations

ASTER: Advanced spaceborne thermal emission and reflection radiometer; AIS: AIDS indicator survey; AIDS: Acquired immune deficiency syndrome; DHS: Demographic health survey; ESRI: Environmental system research institute; GDEM: Global digital elevation model; GIS: Geographic information system; GlobCover: Global cover; GPS: Global positioning system; ITN: Insecticide treated net; LCCS: Land cover classification system; MDG: Millennium development goals; MERG: Monitoring and evaluation reference group; MERIS: MEdium resolution imaging spectrometer; METI: Ministry of economy trade and industry; MIS: Malaria Indicator Survey; MoHSS: Ministry of health and social services; NASA: National aeronautics and space administration; RBM: Roll back malaria; UN: United nations; WHO: World health organization.

## Competing interests

The authors declare that they have no competing interests.

## Authors' contributions

VAA was responsible for study design, data cleaning, analysis, interpretation, drafting and production of the final manuscript. PU contributed to the data assembly, cleaning and contributed to final manuscript. JW was responsible for study design, analysis, interpretation and production of the final manuscript. AMN, PMA and RWS were responsible for overall scientific management, analysis, interpretation and preparation of the final manuscript. All authors read and approved the final manuscript.

## Funding

VAA is supported by the Wellcome Trust Fellowship (#090633). AMN is supported by the Wellcome Trust as an Intermediate Research Fellow (#095127). RWS is supported by the Wellcome Trust as Principal Research Fellow (#079080). This work was partly funded a grant from the Namibia Ministry of Health and Social Services-Global Fund Programme and a Wellcome Trust Major Overseas Programme grant to the KEMRI/Wellcome Trust Research Programme (#092654). This work forms part of the output of the Malaria Atlas Project (MAP, http://www.map.ox.ac.uk), principally funded by the Wellcome Trust, UK. VAA, AMN and RWS also acknowledge support from the Kenya Medical Research Institute. The funders played no role in the study design, data collection and analysis, decision to publish, or preparation of the manuscript.

## References

[B1] Bullen N Moon G Jones K Defining localities for health planning: a GIS approach Soc Sci Med 1996 42 6 801 816 10.1016/0277-9536(95)00180-8 8778994

[B2] Shortt NK Moore A Coombes M Wymer C Defining regions for locality health care planning: a multidimensional approach Soc Sci Med 2005 60 12 2715 2727 10.1016/j.socscimed.2004.11.016 15820582

[B3] Aday LA Andersen R A framework for the study of access to medical care Heal Serv Res 1974 9 208 220 PMC1071804 4436074

[B4] Joseph AE Phillips DR Accessibility and utilization: Geographical perspectives on health care deliver 1984 London, UK: Harper & Row

[B5] Gulliford M Figueroa-Munoz J Morgan M Hughes D Gibson B Beech R Hudson M What does access to health care mean? J Health Serv Res Policy 2002 7 186 188 10.1258/135581902760082517 12171751

[B6] Guagliardo MF Spatial accessibility of primary care: concepts, methods and challenges Int J Health Geogr 2004 3 1 3 10.1186/1476-072X-3-3 14987337 PMC394340

[B7] Haas JS Phillips KA Sonneborn D McCulloch CE Baker LC Kaplan CP Perez-Stable EJ Liang SY Variation in access to health care for different racial/ethnic groups by the racial/ethnic composition of an individual's county of residence Med Care 2004 42 7 707 714 10.1097/01.mlr.0000129906.95881.83 15213496

[B8] Martin D Jordan H Roderick P Taking the bus: incorporating public transport timetable data into health care accessibility modelling Environment and Planning A 2008 40 10 2510 2525 10.1068/a4024

[B9] Robertson R Burge P The impact of patient choice of provider on equity: analysis of a patient survey J Health Serv Res Policy 2011 16 (suppl_1):22 28 21460346 10.1258/jhsrp.2010.010084

[B10] Whitty CJ Chandler C Ansah E Leslie T Staedke SG Deployment of ACT antimalarials for treatment of malaria: challenges and opportunities Malar J 2008 7 (Suppl 1):S7 10.1186/1475-2875-7-S1-S7 PMC2604871 19091041

[B11] Black RE Morris SS Bryce J Where and why are 10 million children dying every year? Lancet 2003 361 9376 2226 2234 10.1016/S0140-6736(03)13779-8 12842379

[B12] Chilundo B Sundby J Aanestad M Analysing the quality of routine malaria data in Mozambique Malar J 2004 3 3 10.1186/1475-2875-3-3 14998435 PMC395838

[B13] Guerra CA Gikandi PW Tatem AJ Noor AM Smith DL Hay SI Snow RW The limits and intensity of Plasmodium falciparum transmission: implications for malaria control and elimination worldwide PLoS Med 2008 5 2 e38 10.1371/journal.pmed.0050038 18303939 PMC2253602

[B14] Hay SI Guerra CA Gething PW Patil AP Tatem AJ Noor AM Kabaria CW Manh BH Elyazar IR Brooker S A world malaria map: plasmodium falciparum endemicity in 2007 PLoS Med 2009 6 3 e1000048 19323591 10.1371/journal.pmed.1000048 PMC2659708

[B15] Cromley EK McLafferty SL GIS and public healt 2002 New York: Guilford Press

[B16] Agyepong IA Kangeya-Kayonda J Providing practical estimates of malaria burden for health planners in resource-poor countries AmJTrop Med Hyg 2004 71 2 Suppl 162 167 15331833

[B17] Cibulskis RE Bell D Christophel EM Hii J Delacollette C Bakyaita N Aregawi MW Estimating trends in the burden of malaria at country level AmJTrop Med Hyg 2007 77 6 Suppl 133 137 18165485

[B18] The health mapper http://gis.emro.who.int/PublicHealthMappingGIS/HealthMapper.aspx

[B19] Noor AM Alegana VA Gething PW Snow RW A spatial national health facility database for public health sector planning in Kenya in 2008 Int J Health Geogr 2009 8 13 10.1186/1476-072X-8-13 19267903 PMC2666649

[B20] The AfriPop project http://www.clas.ufl.edu/users/atatem/index_files/AfriPop.htm

[B21] Haynes R Bentham C Lovett A Gale S Effects of distances to hospital and GP surgery on hospital inpatient episodes, controlling for needs and provision Soc Sci Med 1999 49 425 433 10.1016/S0277-9536(99)00149-5 10414825

[B22] Tanser F Hosegood V Benzler J Solarsh G New approaches to spatially analyse primary health care usage patterns in rural South Africa Trop Med Int Health 2001 6 10 826 838 10.1046/j.1365-3156.2001.00794.x 11679131

[B23] Buor D Analysing the primacy of distance in the utilization of health services in the Ahafo-Ano South district, Ghana 2003 18 Wiley 293 311 10.1002/hpm.729 14727709

[B24] Noor AM Zurovac D Hay SI Ochola SA Snow RW Defining equity in physical access to clinical services using geographical information systems as part of malaria planning and monitoring in Kenya Trop Med Int Health 2003 8 10 917 926 10.1046/j.1365-3156.2003.01112.x 14516303 PMC2912492

[B25] Gething PW Noor AM Zurovac D Atkinson PM Hay SI Nixon MS Snow RW Empirical modelling of government health service use by children with fevers in Kenya Acta Trop 2004 91 3 227 237 10.1016/j.actatropica.2004.05.002 15246929 PMC3166847

[B26] Noor AM Amin AA Gething PW Atkinson PM Hay SI Snow RW Modelling distances travelled to government health services in Kenya Trop Med Int Health 2006 11 2 188 196 10.1111/j.1365-3156.2005.01555.x 16451343 PMC2912494

[B27] Martin D Wrigley H Barnett S Roderick P Increasing the sophistication of access measurement in a rural healthcare study Health & Place 2002 8 3 13 10.1016/S1353-8292(01)00031-4 11852259

[B28] Schuurman N Fiedler RS Grzybowski SCW Grund D Defining rational hospital catchments for non-urban areas based on travel-time Int J Health Geogr 2006 5 43 10.1186/1476-072X-5-43 17018146 PMC1617091

[B29] Walsh SJ Page PH Gesler WM Normative models and healthcare planning: network-based simulations within a geographic information system environment Health Serv Res 1997 32 2 243 260 9180618 PMC1070185

[B30] Jordan H Roderick P Martin D Barnett S Distance, rurality and the need for care: access to health services in South West England Int J Heal Geogr 2004 3 1 21 10.1186/1476-072X-3-21 PMC524184 15456514

[B31] Tanser F Gijsbertsen B Herbst K Modelling and understanding primary health care accessibility and utilization in rural South Africa: an exploration using a geographical information system Soc Sci Med 2006 63 3 691 705 10.1016/j.socscimed.2006.01.015 16574290

[B32] Apparicio P Abdelmajid M Riva M Shearmur R Comparing alternative approaches to measuring the geographical accessibility of urban health services: distance types and aggregation-error issues Int J Health Geogr 2008 7 7 10.1186/1476-072X-7-7 18282284 PMC2265683

[B33] Bailey CT Gatrell CA Interactive Spatial Data Analysi 1995 Essex, England: Longman Scientific & Technical

[B34] Talen E Anselin L Assessing spatial equity: an evaluation of measures of accessibility to public playgrounds Environ Planning A 1998 30 4 595 613 10.1068/a300595

[B35] Akin JS Hutchinson P Health-care facility choice and the phenomenon of bypassing Health Policy Plan 1999 14 2 135 151 10.1093/heapol/14.2.135 10538717

[B36] MoHSS Ministry of Health and Social Services (MoHSS) RoN Health and Social services system review 2008 Windhoek

[B37] MoHSS, ICF Macro Namibia Health Facility Census (HFC) 2009 2010 Windhoek, Namibia: Windhoek, Namibia. MoHSS and ICF Macro 585

[B38] El Obeid S Mendelsohn JM Lejars M Forster N Brulé G Health in Namibia: Progress and challenges 2001 Windhoek, Namibia: Research and Information Services of Namibia (RAISON) Available at: http://www.raison.com.na/page_htm_raison/downloads.html [Accessed 17 May 2011]

[B39] Zere E Mbeeli T Shangula K Mandlhate C Mutirua K Tjivambi B Kapenambili W Technical efficiency of district hospitals: evidence from Namibia using data envelopment analysis Cost Eff Resour Alloc 2006 4 5 10.1186/1478-7547-4-5 16566818 PMC1524815

[B40] Ministry of Health and Social Services (MoHSS) [Bethesda] MaHS Namibia national health accounts 2001/02-2006/07 2008 Windhoek, Namibia

[B41] Google Earth http://www.google.co.uk/intl/en_uk/earth/

[B42] The GeoNames geographical database http://www.geonames.org/search.html?q=&country=NA

[B43] Briggs DJ Gulliver J Fecht D Vienneau DM Dasymetric modelling of small-area population distribution using land cover and light emissions data Remote Sens Environ 2007 108 4 451 466 10.1016/j.rse.2006.11.020

[B44] Bhaduri B Bright E Coleman P Urban M LandScan USA: a high-resolution geospatial and temporal modeling approach for population distribution and dynamics GeoJournal 2007 69 1 103 117 10.1007/s10708-007-9105-9

[B45] Linard C Gilbert M Tatem A Assessing the use of global land cover data for guiding large area population distribution modelling GeoJournal 2011 76 5 525 538 10.1007/s10708-010-9364-8 PMC3617592 23576839

[B46] Mendelsohn JM Roberts CS An environmental atlas and profile of Capriv 1997 Windhoek: Ministry of Environment and Tourism, Windhoek

[B47] Mendelsohn JM El Obeid S A preliminary profile of Kavango region Windhoek.: Namibia Nature Foundation 2001

[B48] Mendelsohn JM El Obeid S Roberts CS A profile of north-central Namibia 2000 Windhoek: Gamsberg MacMillan

[B49] Water Resource Programme Analysis of Present and Future Water demand in Namibia: Namibia Water Resources Management Review 2007 Windhoek: Windhoek Consulting Engineers

[B50] Land Cover Classification System (LCCS): Classification Concepts and User Manual http://www.fao.org/docrep/003/x0596e/x0596e00.htm

[B51] Namibia 5-Year Age/Sex Population Estimates 2000-2010: Second Administrative Level Divisions http://www.hivspatialdata.net/xslview.aspx?docid=690&country=Namibia

[B52] RBM-MERG Roll Back Malaria-Monitoring and Evaluation Resource Group: Core Household Questionnaire 2005 Calverton, Maryland

[B53] Demographic and Health Surveys http://www.measuredhs.com

[B54] Spatial Data Download Namibia http://www.diva-gis.org/gData

[B55] Digital atlas of Namibia http://www.uni-koeln.de/sfb389/e/e1/download/atlas_namibia/e1_download_physical_geography_e.htm

[B56] GlobCover 2009 (Global Land Cover Map) http://ionia1.esrin.esa.int/

[B57] Ray N Ebener S AccessMod 3.0: computing geographic coverage and accessibility to health care services using anisotropic movement of patients Int J Heal Geogr 2008 7 1 63 10.1186/1476-072X-7-63 PMC2651127 19087277

[B58] The Government of the Republic of Namibia The Road Traffic and Transport Regulations, 2001 Government Notice No No161 of 2002, published in Government Gazette No2815 of 26 September 2002 2002 Namibia: Ministry of Transport 323 324

[B59] Tobler W Three presentations on geographical analysis and modeling: National Center for Geographic Information and Analysis Technical report 93-1 1993 Santa Barbara, CA93106-4060: University of California, Santa Barbara

[B60] Pinheiro J Bates D Mixed Effects Models in S and S-Plus 2002 Springer

[B61] Gething PW Kirui VC Alegana VA Okiro EA Noor AM Snow RW Estimating the number of paediatric fevers associated with malaria infection presenting to Africa's public health sector in 2007 PLoS Med 2010 7 7 e1000301 10.1371/journal.pmed.1000301 20625548 PMC2897768

[B62] Ensor T Cooper S Overcoming barriers to health service access: influencing the demand side Health Policy and Planning 2002 19 2 69 79 10.1093/heapol/czh009 14982885

[B63] Bi-Logistic growth http://phe.rockefeller.edu/Bi-Logistic/

[B64] Gershenfeld NA The Nature of Mathematical Modelin 1999 Cambridge, UK: Cambridge University Press

[B65] Leonard K Mliga GR Mariam DH Bypassing health centers in Tanzania: Revealed preferences for observable and unobservable qualit 2002 Columbia University, Department of Economics

[B66] Gething P Atkinson P Noor A Gikandi P Hay S Nixon M A local space-time kriging approach applied to a national outpatient malaria dataset Comput Geosci 2007 33 10 1337 1350 10.1016/j.cageo.2007.05.006 19424510 PMC2677680

[B67] Moisi JC Nokes DJ Gatakaa H Williams TN Bauni E Levine OS Scott JA Sensitivity of hospital-based surveillance for severe disease: a geographic information system analysis of access to care in Kilifi district, Kenya Bull World Health Organ 2011 89 2 102 111 10.2471/BLT.10.080796 21346921 PMC3040379

[B68] Al-Taiar A Jaffar S Assabri A Al-Habori M Azazy A Al-Gabri A Al-Ganadi M Attal B Whitty CJ Who develops severe malaria? Impact of access to healthcare, socio-economic and environmental factors on children in Yemen: a case-control study Trop Med Int Health 2008 13 6 762 770 10.1111/j.1365-3156.2008.02066.x 18410250

[B69] O'Meara WP Noor A Gatakaa H Tsofa B McKenzie FE Marsh K The impact of primary health care on malaria morbidity - defining access by disease burden Trop Med Int Health 2009 14 1 29 35 10.1111/j.1365-3156.2008.02194.x 19121148 PMC2658804

[B70] Okwaraji Y Cousens S Berhane Y Mulholland K Edmond K Effect of geographical access to health facilities on child mortality in rural Ethiopia: a community based cross sectional study PLoS ONE 2011 Submitted for review 10.1371/journal.pone.0033564 PMC3299799 22428070

[B71] Kindermans J-M Vandenbergh D Vreeke E Olliaro P D'Altilia J-P Estimating antimalarial drugs consumption in Africa before the switch to artemisinin-based combination therapies (ACTs) Malar J 2007 6 1 91 10.1186/1475-2875-6-91 17623092 PMC1948000

[B72] Okiro E Alegana V Noor A Mutheu J Juma E Snow R Malaria paediatric hospitalization between 1999 and 2008 across Kenya BMC Med 2009 7 1 75 10.1186/1741-7015-7-75 20003178 PMC2802588

[B73] Gething PW Noor AM Gikandi PW Hay SI Nixon MS Snow RW Atkinson PM Developing geostatistical space-time models to predict outpatient treatment burdens from incomplete national data Geogr Anal 2008 40 2 167 188 10.1111/j.1538-4632.2008.00718.x 19325928 PMC2660576

[B74] Snow RW Eckert E Teklehaimanot A Estimating the needs for artesunate-based combination therapy for malaria case-management in Africa Trends Parasitol 2003 19 8 363 369 10.1016/S1471-4922(03)00168-5 12901938

[B75] Okiro E Snow R The relationship between reported fever and Plasmodium falciparum infection in African children Malar J 2010 9 1 99 10.1186/1475-2875-9-99 20398428 PMC2867992

[B76] Okiro EA Alegana VA Noor AM Snow RW Changing malaria intervention coverage, transmission and hospitalization in Kenya Malar J 2010 9 285 10.1186/1475-2875-9-285 20946689 PMC2972305

[B77] Elmardi KA Noor AM Githinji S Abdelgadir TM Malik EM Snow RW Self-reported fever, treatment actions and malaria infection prevalence in the northern states of Sudan Malar J 2011 10 128 10.1186/1475-2875-10-128 21575152 PMC3115918

